# Whole Tumor Heterogeneity Topography (WTHT)—A New Approach for Assessment of Intratumor Mutational Heterogeneity in Colorectal Adenomas

**DOI:** 10.3390/ijms27156547

**Published:** 2026-07-23

**Authors:** Tereza Halkova, Lucia Hodasova Pauerova, Karolina Blechova, Tereza Benesova, Tomas Grega, Nadija Brodyuk, Eva Traboulsi, Katerina Hejcmanova, Ondrej Ngo, Jan Bures, Stepan Suchanek, Lucie Benesova

**Affiliations:** 1 Centre for Applied Genomics of Solid Tumors (CEGES), Genomac Research Institute, Drnovská 1112/60, 161 00 Prague, Czech Republic; thalkova@genomac.cz (T.H.); lhodasovapauerova@genomac.cz (L.H.P.); kblechova@genomac.cz (K.B.); tbenesova@genomac.cz (T.B.); lbenesova@genomac.cz (L.B.); 2 Department of Internal Medicine, 1st Faculty of Medicine, Military University Hospital Prague, Charles University, Prague, U Vojenske Nemocnice 1200, 169 02 Prague, Czech Republic; gregatom@uvn.cz (T.G.); nadiya.brodyuk@uvn.cz (N.B.); 3 Department of Gastrointestinal Oncology, 1st Faculty of Medicine, Military University Hospital Prague, Charles University, Prague, U Vojenske Nemocnice 1200, 169 02 Prague, Czech Republic; bures.jan@uvn.cz; 4 Department of Pathology, Military University Hospital Prague, U Vojenske Nemocnice 1200, 169 02 Prague, Czech Republic; eva.traboulsi@uvn.cz; 5 Institute of Biostatistics and Analyses, Faculty of Medicine, Masaryk University, 601 77 Brno, Czech Republic; hejcmanova@iba.muni.cz (K.H.); ngo@iba.muni.cz (O.N.); 6 Institute of Health Information and Statistics of the Czech Republic, 128 00 Prague, Czech Republic

**Keywords:** intratumor mutation heterogeneity, index lesion, colorectal adenoma, mutation clone, whole tumor heterogeneity topography

## Abstract

Intratumor heterogeneity (ITH) of premalignant colorectal lesions is an important area of research for understanding the diverse biological behavior of colorectal cancer (CRC). Accurate assessment of ITH depends substantially on the sampling strategy used. We present a novel methodological approach termed whole tumor heterogeneity topography (WTHT) and compare it with three previously described sampling strategies: whole tumor homogenization, macrodissection of selected tumor regions, and multisampling. A cohort of 184 advanced precancerous colorectal lesions was processed into paraffin blocks, and the entire tumor mass was systematically divided into equally sized samples (~10 mm^3^). DNA was isolated from each sample separately and analyzed for hotspot mutations in *APC*, *KRAS*, *BRAF*, *PIK3CA*, and *TP53*. ITH was quantified using mutation variance and a newly introduced Heterogeneity Grade (HG). Results obtained by WTHT were statistically and graphically compared with three other modeled sampling strategies. Significant differences were observed among the analyzed sampling approaches. Macrodissection showed the greatest deviation from WTHT, indicating substantial sampling bias, whereas multisampling produced the closest results. WTHT enabled precise quantification and spatial mapping of mutational clones across the entire lesion. WTHT combined with HG provides a robust and reproducible framework for comprehensive assessment of ITH in colorectal adenomas.

## 1. Introduction

Colorectal cancer (CRC) is a heterogeneous, multifactorial disease resulting from a combination of genetic and environmental factors, where even individuals with pathologically similar tumors often have different courses of the disease and survival. One of the sources of this so-called intertumoral heterogeneity [1] is its localization, where CRC in the right colon (proximal colon from cecum to transverse colon), left colon (distal colon from splenic flexure to rectosigmoid), and rectum has different clinical manifestations, histological characteristics, and is associated with different responses to therapy and overall prognosis. This has been shown in smaller studies [2,3,4] and clearly confirmed in a study with almost 58 thousand patients [5]. Possible explanations include the different embryonic origin of the proximal colon from the distal colon and rectum, their different physiological functions [6], or exposure to different bacterial populations, nutrients, and bile acids on both sides of the intestine [7]. From a molecular genetic perspective, it has been found that more than 1000 genes are expressed differently in the right and left colon [8].

Another source of intertumoral heterogeneity in CRC is the different pathways leading to its development. Currently, 2 main pathways of carcinogenesis are distinguished in sporadic (non-hereditary) CRC—the chromosomal instability (CIN) pathway and the serrated pathway [9,10]. The CIN pathway accounts for most CRC (~80%) and in general corresponds to the model of the adenoma–carcinoma sequence, also known as the Fearon–Vogelstein model of colorectal carcinogenesis first described in 1990 [11]. This pathway is widely accepted to this day [12,13,14,15] and is presented as a multistep process beginning with the formation of a benign precancerous lesion (adenoma), which can gradually transform into an invasive carcinoma. This process can last up to 15 years [16] and is accompanied by the inactivation of tumor suppressor genes and activation of proto-oncogenes, mainly *APC*, *TP53*, *PIK3CA*, and *KRAS* [17]. It has gradually become clear that the accumulation of typical mutations is coupled with chromosomal instability (CIN), which is characterized by chromosome number imbalances and loss of heterozygosity. That is why this pathway is currently referred to as CIN. CRC develops in the CIN pathway through so-called conventional adenomas, which are histologically classified as tubular (TA), villous (VA), or tubulovillous (TVA) if both components are present. They are further divided according to the degree of dysplasia into low-grade dysplasia (LGD) or high-grade dysplasia (HGD) [9].

**Figure 1:**
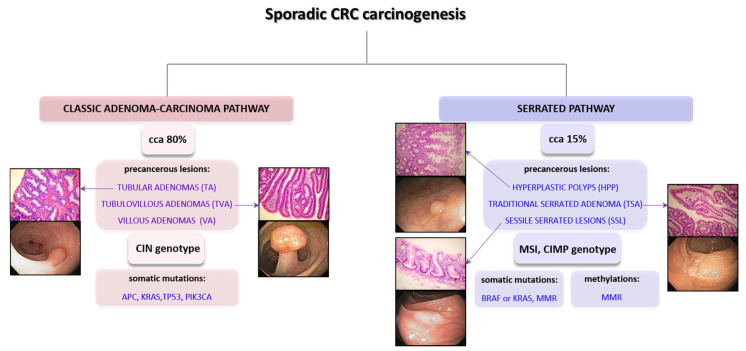
Scheme of sporadic colorectal carcinogenesis. Representative macroscopic images from colonoscopy and corresponding microscopic images of histological sections are shown for individual types of precancerous colorectal lesions. Histological sections were stained with hematoxylin and eosin and were obtained from the archive of the Department of Pathology (magnification ×100), University Military Hospital Prague. Colonoscopic images were adapted from ref. [28].

About 15% of CRC arises through the alternative serrated pathway. The serrated pathway includes lesions with a histologically typical serrated appearance. Unlike conventional adenomas that protrude above the intestinal mucosa, serrated lesions are flat. These include hyperplastic polyps (HPP), which can progress to sessile serrated lesions (SSL) or traditional serrated adenoma (TSA) and finally to CRC. All the above serrated lesions can also arise de novo [18,19,20]. A typical DNA mutation associated with the serrated pathway is a mutation in the *BRAF* proto-oncogene, as well as a mutation in the *KRAS* proto-oncogene in part of the serrated adenomas. CRCs generated by this pathway do not show CIN but are typically characterized by the CpG island methylator phenotype (CIMP), involving widespread aberrant CpG island methylation that affects gene regulation, including frequent hypermethylation of promoter regions of tumor suppressor genes, leading to transcriptional silencing [4,21,22,23,24]. Furthermore, CRCs formed by the serrated pathway are characterized by microsatellite instability (MSI); i.e., different cells in the colorectal tissue carry different numbers of short repetitive DNA sequences (so-called microsatellites). MSI is a manifestation of a dysfunctional Mismatch Repair (MMR) system that ensures DNA repair during replication. Its dysfunction occurs due to the inactivation of MMR genes (e.g., *MLH1*, *MSH2*, *MSH6*, *PMS1*, *PMS2*) through somatic deletions, point mutations, or epigenetic silencing [25,26]. This results in the accumulation of DNA mutations, among others, in tumor suppressor genes and proto-oncogenes. MMR genes have also been found to play a role in cell cycle control and apoptosis. All of this contributes to the risk of tumorogenesis [27]. The key features of both pathways of sporadic CRC development are summarized in Figure 1.

In addition to the above-mentioned intertumoral heterogeneity naturally resulting from the localization of CRC and the pathway of its origin, a separate important term in this regard is the so-called intratumor heterogeneity (ITH). ITH has been recognized since the 1980s and is understood as the presence of distinct clonal subpopulations of tumor cells within a single tumor with variable genetic changes and phenotypic traits [29]. From a clinical point of view, such individual cell populations may have different effects on tumor behavior—for example, they may differ in growth rate, ability to metastasize [30,31,32], and respond differently to a given type of therapy [33,34,35]. The presence of distinct cell populations stems from the presence of different genetic changes—mutations—within a tumor. As tumor cells divide, random mutations accumulate and are passed on to daughter cells, resulting in the presence of many different mutational subclones [36].

With the advent of next-generation sequencing (NGS) technologies, the presence of ITH has been widely confirmed across different tumor types (e.g., [37,38]). Despite this, only a limited number of studies have examined in detail the distribution of individual mutations within colorectal cancer [39,40,41,42], and studies focusing on adenomas, early stages of carcinogenesis where this heterogeneity arises, are even less common. Existing data are based on relatively small cohorts; e.g., 8 high-grade adenomas [43], 1 adenoma [44], 48 early polyps [45], 10 early colorectal tumors [46], and 90 precancerous colorectal lesions [47]. However, none of the above studies contain a detailed characterization of ITH or individual mutation clones in the entire adenoma—at most in its representative part(s)—so the true picture of ITH may be distorted.

**Figure 2:**
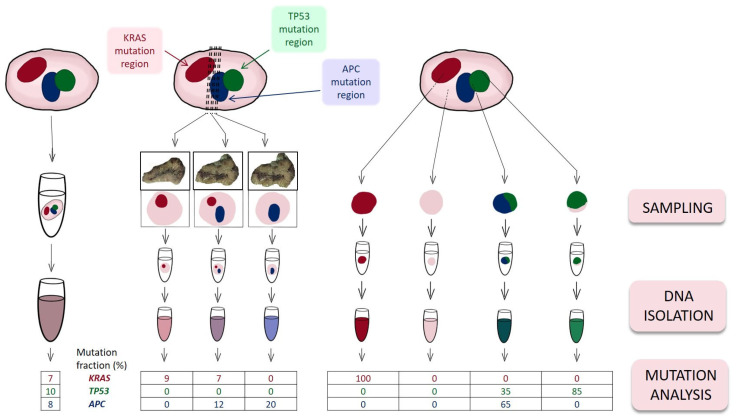
Three sampling strategies used for the study of ITH. From the left: first sampling strategy = homogenization of the entire tumor mass into one sample, next sampling strategy = macrodissection of selected tumor regions (here of three sections), final sampling strategy = multisampling from multiple areas (here from four areas).

The inability to obtain a comprehensive view of ITH primarily results from the sampling of tumor tissue for its study. Currently, three main sampling approaches are used, which are shown in Figure 2. The first is the homogenization of the entire tumor mass into one sample, from which the DNA containing all the mutations present in the tumor is then isolated. Although this approach appears rational, it does not provide information about the spatial localization of individual mutational clones, their representation in different parts of the tumor, or their evolution. As a result, this strategy is rarely used in practice [48,49]. The second approach is macrodissection of selected tumor regions; for example, from several representative FFPE tissue sections [45,47,50]. When DNA from individual sections is isolated and analyzed separately, this strategy can provide insight into ITH, but only in selected areas of the tumor tissue. A third approach is multisampling; i.e., macrodissection of tumor tissue from several regions of the tumor mass [39,40,43,46]. A special variant of this method is single-cell microdissection from different locations of the tumor tissue [42]. This approach provides useful information about ITH in different parts of the tumor, but it cannot be applied to the entire tumor mass, as most of the tumor remains unanalyzed; thus, the idea of the number of mutations and the development of mutational clones is necessarily distorted. In summary, none of the three approaches above allows comprehensive assessment of ITH in the entire tumor, and the second and third approaches also fail to accurately quantify the total mutational load within the tumor.

Currently, mutational analysis of ITH is performed almost exclusively using NGS, either by whole-exome sequencing (WES) or targeted sequencing of panels comprising tens to hundreds of cancer-associated genes. These approaches allow detailed characterization of the tumor mutational spectrum, including the detection of subclonal populations, and thus represent a useful tool for studying tumor evolution. On the other hand, they are associated with a number of limitations. These include, in particular, time-consuming library preparation, higher requirements for DNA quality and quantity, the need for sophisticated bioinformatic data analysis and interpretation, and the still relatively high financial cost. These factors limit the routine use of complex NGS approaches in clinical practice or in the analysis of large sample cohorts. Despite this technologically advanced and comprehensive NGS analysis, a limited number of key genes, such as *APC*, *KRAS*, *PIK3CA*, *TP53,* and *BRAF*, continue to retain essential biological and clinical significance. Mutations in these genes have been repeatedly confirmed as key events in the initiation and progression of CRC and often dominate among individual tumor subclones [47,51]. This suggests that the biological basis of CRC remains driven by a relatively small number of key genetic alterations. From this perspective, targeted analysis of these critical genes may be more effective than comprehensive whole-exome profiling, particularly when the goal is rapid, cost-effective, and clinically applicable tumor characterization.

**Figure 3:**
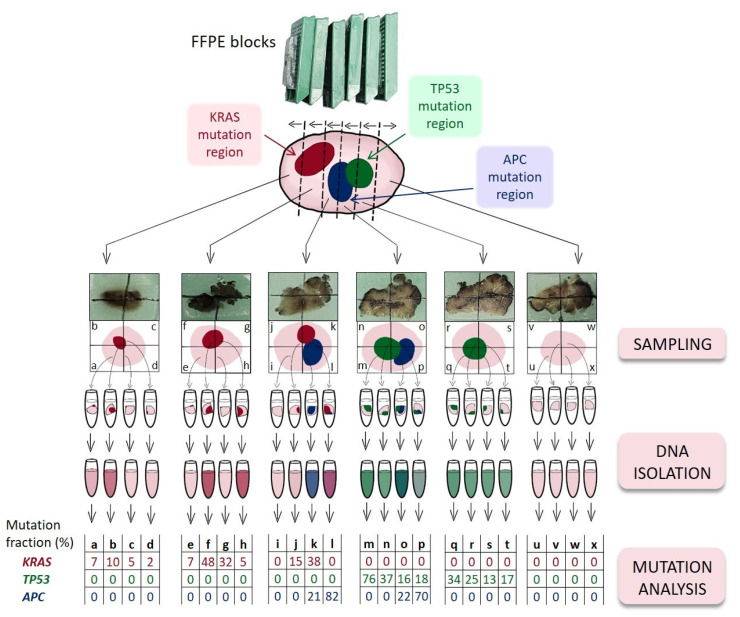
WTHT approach for studying ITH. Schematic illustration of the WTHT methodology based on systematic division of the entire tumor mass into small-volume units labeled a–x (~10 mm^3^), followed by separate DNA isolation and mutation analysis of individual samples. The approach enables identification of mutational clones, their quantification, and assessment of their spatial distribution in different areas covering the entire lesion.

In this publication, we build on all the above-mentioned findings and demonstrate that ITH can also be studied using the opposite approach, by prioritizing higher-quality sampling over the quantity of analyzed genetic loci. In an effort to comprehensively map ITH in the entire adenoma, we introduce the method “Whole tumor heterogeneity topography” (WTHT), which is based on processing the entire tumor mass into samples of approximately 10 mm^3^ and subsequent analysis of the most frequently mutated genes in these samples for assessing the presence of mutational clones, their spatial distribution, and quantitative representation. This approach is clearly illustrated in Figure 3.

At the same time, we introduce a novel quantitative parameter, the heterogeneity grade (HG), which enables numerical assessment of the degree of ITH. HG represents a simple and reproducible tool for comparing ITH both between samples and between studies.

## 2. Results

### 2.1. General Characteristics of the Lesion Set

The lesions were categorized into groups according to histopathological types and sorted according to dysplasia grade. The study included 9 HPP, 34 TSA, 43 TA LGD, 12 TA HGD, 3 TA HGD with intramucosal carcinoma structures (=TA HGD ca), 60 TVA LGD, 12 TVA HGD, and 11 TVA HGD with intramucosal carcinoma structures (=TVA HGD ca). The occurrence of individual histological types in different sections of the intestine is shown in the heatmap in Supplementary Figure S1. The primary dataset is provided in Supplementary Table S1.

#### 2.1.1. Prevalence of Mutations in Individual Sections of the Colon

**Figure 4:**
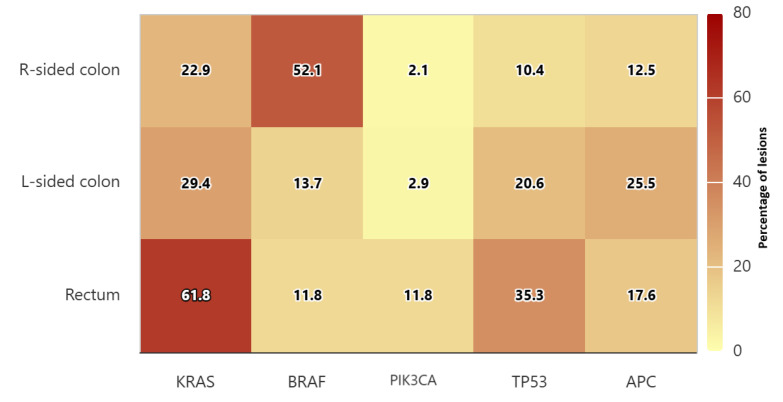
The prevalence of tested mutations depending on the localization of the lesion.

The prevalence of tested mutations depending on the localization of lesions in individual sections of the colon is shown in Figure 4. There is an obvious increase in the incidence of lesions with a *KRAS*, *PIK3CA,* or *TP53* mutation from the right colon to the rectum and, conversely, a decrease in the incidence of lesions with a *BRAF* mutation in the same direction. Lesions harboring *APC* mutations were predominantly localized in the left colon.

Differences in mutation prevalence according to anatomical localization were significant for *KRAS, BRAF,* and *TP53,* whereas *APC* and *PIK3CA* showed no significant differences after correction for multiple testing. Results are listed in Supplementary Table S2.

#### 2.1.2. Prevalence of Mutations Depending on the Histological Type of Lesion

**Figure 5:**
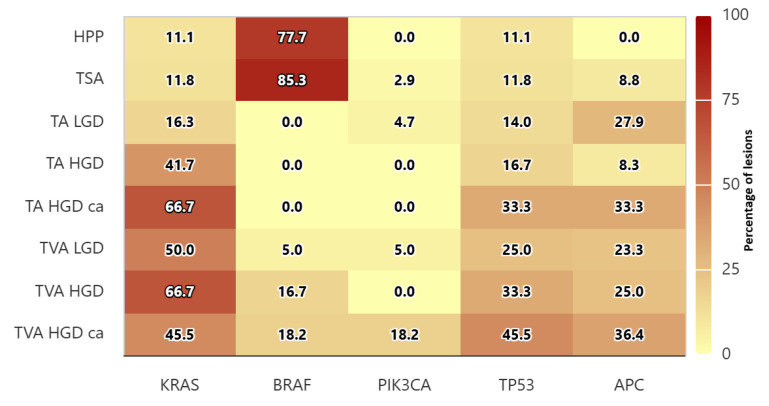
The prevalence of tested mutations depending on the histological type of lesion.

The prevalence of tested mutations depending on the histological type of lesion is shown in Figure 5. The presence of a *BRAF* mutation is typical for serrated lesions (HPP, TSA), while the presence of a *KRAS* mutation is characteristic of conventional adenomas (TA, TVA). Interestingly, *BRAF* mutation was not found in any tubular adenoma. *TP53* mutations were detected in all histological types of precancerous colorectal lesions, and their incidence increases with an increasing degree of dysplasia. In general, the presence of *TP53* mutation is characteristic especially for TVA, where it occurs in approximately one third of the cases (24/83; 28.9%). *APC* mutations occur in all types of adenomas (TSA, TA, TVA) but were not found in any HPP. *PIK3CA* mutations were detected least frequently, occurring in only 1/43 (2.3%) serrated lesions, 2/58 (3.4%) TAs, and 5/83 (6.0%) TVAs.

Differences in mutation prevalence among histological groups were significant for *KRAS* and *BRAF*, but not for *TP53*, *APC,* or *PIK3CA*. Results are shown in Supplementary Table S2.

### 2.2. ITH vs. Mutation Analysis

**Figure 6:**
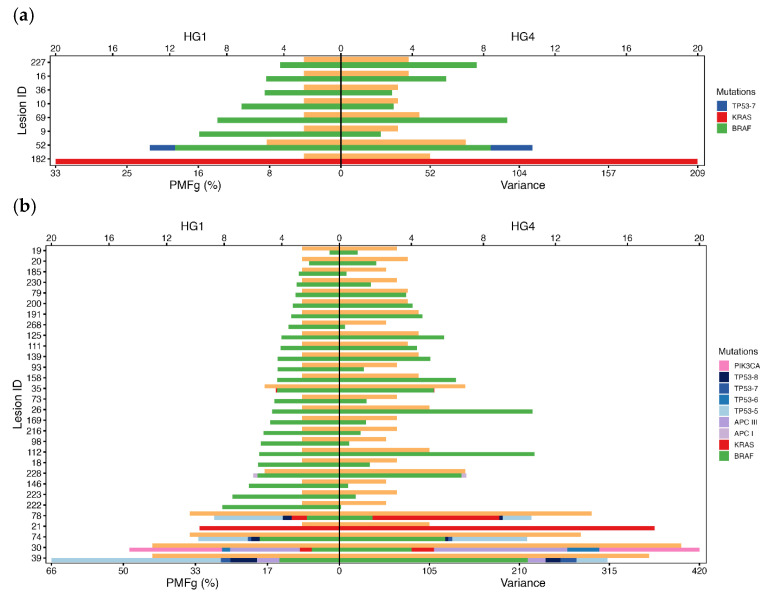
Representation of mutation clones found in serrated lesions. Left side: $\bar{PMFg}$ (colored) and HG1 (orange). Right side: Variance (colored) and HG4 (orange). Lesions are sorted by increasing $\bar{PMFg}$, and the order is preserved on both sides of the graph. (**a**) representation of mutation clones in 8 HPP. 1 HPP (ID 186), in which none of the tested mutation was detected, is not included in the figure; (**b**) representation of mutation clones in 30 TSA. Four TSA (ID 49, 66, 92, 253), in which none of the tested mutation was detected, are not included in the figure.

**Figure 7:**
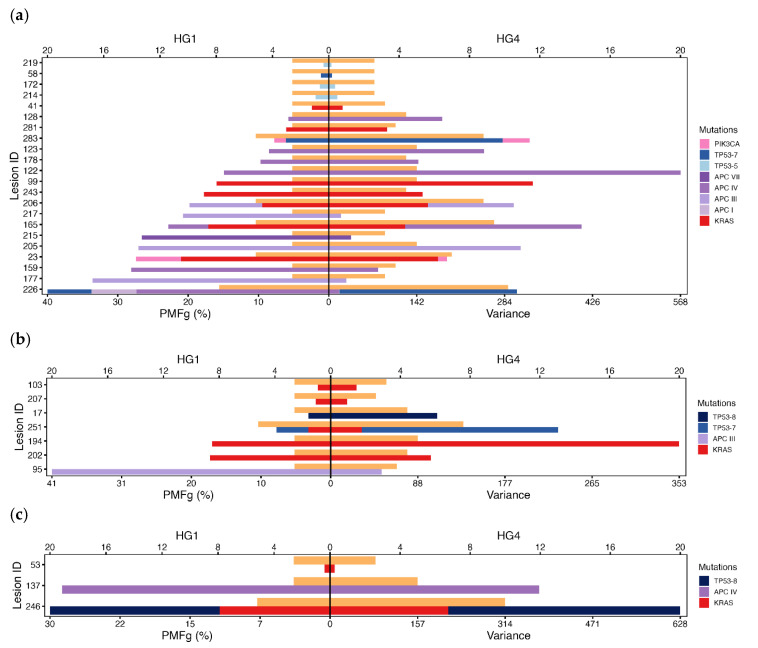
Representation of mutation clones found in tubular adenomas. **Left side**: $\bar{PMFg}$ (colored) and HG1 (orange). **Right side**: Variance (colored) and HG4 (orange). Lesions are sorted by increasing $\bar{PMFg}$, and the order is preserved on both sides of the graph. (**a**) representation of mutation clones in 22 TA LGD. A total of 21 TA LGD (ID 24, 28, 29, 31, 32, 57, 60, 71, 140, 187, 190, 196, 198, 201, 213, 218, 231, 232, 252, 260, 292), in which none of the tested mutation was detected, are not included in the figure; (**b**) representation of mutation clones in 7 TA HGD. Five TA HGD (ID 2, 154, 170, 181, 248), in which none of the tested mutation was detected, are not included in the figure; (**c**) representation of mutation clones in 3 TA HGD ca. There were no TA HGD ca without mutation in the lesion set.

**Figure 8:**
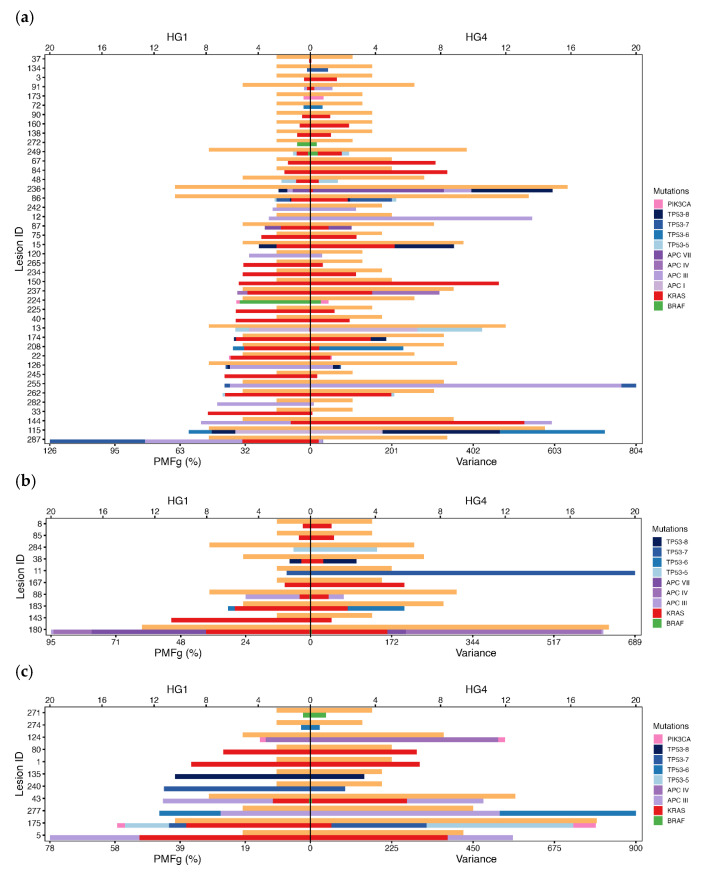
Representation of mutation clones found in tubulovillous adenomas. **Left side**: $\bar{PMFg}$ (colored) and HG1 (orange). **Right side**: Variance (colored) and HG4 (orange). Lesions are sorted by increasing $\bar{PMFg}$, and the order is preserved on both sides of the graph. (**a**) representation of mutation clones in 42 TVA LGD. A total of 18 TVA LGD (ID 7, 42, 46, 59, 68, 70, 77, 102, 113, 133, 148, 164, 166, 179, 189, 192, 233, 273), in which none of the tested mutation was detected, are not included in the figure; (**b**) representation of mutation clones in 10 TVA HGD. Two TVA HGD (ID 76, 153), in which none of the tested mutation was detected, are not included in the figure; (**c**) representation of mutation clones in 11 TVA HGD ca. There were no TVA HGD ca without mutation in the lesion set.

Using the WTHT approach, the ITH of each lesion was expressed by the variance of mutation(s) and the HG4 value (right side of the graphs on Figure 6, Figure 7 and Figure 8). For comparison, the simple overall percentage of individual mutations in the entire lesion ($\bar{PMFg}$) and the corresponding HG1 value, obtained by modeled sampling strategy 1, were determined for each lesion (left side of the graphs on Figure 6, Figure 7 and Figure 8).

As the graphs indicate, variance is a key component of the correct calculation of HG, and consequently of ITH assessment. A high $\bar{PMFg}$ value of a certain mutation in a lesion does not automatically mean a high variance. Examples include lesions ID 146 and 222 (Figure 6b), 177 (Figure 7a), and 33 (Figure 8a), where $\bar{PMFg}$ reaches the highest values within the given lesion group, while variance shows below-average values. Conversely, at average $\bar{PMFg}$ values, the variance can reach above-average to very high levels, as observed in lesions ID 26, 112, and 21 (Figure 6b), 122 (Figure 7a), and 12 (Figure 8a).

This effect is even more pronounced in lesions containing multiple mutant clones, where two types of discrepancies can be observed. In the first case, the $\bar{PMFg}$ of all mutant clones is lower than their variance; e.g., lesion ID 30 (Figure 6b), 283 (Figure 7a), and 236 (Figure 8a), or vice versa in the case of lesion ID 287 (Figure 8a). In the second case, the $\bar{PMFg}$ of some mutant clones is lower than their variance, whereas for other mutational clones the opposite pattern is observed; e.g., lesion ID 226 (Figure 7a) and 175 (Figure 8c).

### 2.3. Impact of Sampling Strategy on Mutation Analysis and HG

For 20 randomly selected colorectal lesions consisting of at least 5 FFPE covering all histological types, HG1–4 were calculated, as shown in Table 1.

HG4 was used as the reference against which HG1–HG3 were compared. HG2 showed the greatest deviation from HG4, with a mean bias of −4.93 points (95% bootstrap CI −6.22 to −3.65), a mean absolute error of 4.93 points (95% CI 3.65–6.22), and a mean relative error of 47.1% (95% CI 39.0–55.2%). HG1 also systematically underestimated HG4, with a mean bias of −3.24 points (95% CI −3.96 to −2.49), a mean absolute error of 3.24 points (95% CI 2.49–3.96), and a mean relative error of 32.7% (95% CI 28.4–36.9%). HG3 showed the smallest deviation from HG4, with a mean bias of −1.21 points (95% CI −2.12 to −0.32), a mean absolute error of 1.87 points (95% CI 1.23–2.55), and a mean relative error of 21.1% (95% CI 14.4–28.5%).

Paired comparison using the Wilcoxon signed-rank test confirmed significant differences between each modeled sampling strategy and HG4 (HG1 vs. HG4, adjusted *p* = 0.00021; HG2 vs. HG4, adjusted *p* = 0.00021; HG3 vs. HG4, adjusted *p* = 0.0418). Bootstrap resampling confirmed the stability of the estimated bias and error metrics across all sampling strategies.

The distortion classification showed different error profiles among sampling strategies 1–3, represented by the respective HGs 1–3. For HG1, 75% of lesions showed high distortion, defined as 25–50% relative error, and 25% showed moderate distortion, defined as 10–25% relative error. For HG2, 40% of lesions showed high distortion, 40% showed severe distortion above 50%, and 20% showed moderate distortion. For HG3, 20% of lesions showed low distortion below 10%, 55% showed moderate distortion, 10% showed high distortion, and 15% showed severe distortion.

The proposed HG framework showed high robustness to changes in both variance binning and mutation-count weighting. Across alternative binning strategies, the recalculated HG values remained highly correlated with the original score (Spearman ρ = 0.973–0.998). Likewise, modifying the mutation-count weight had only a negligible effect on HG (Spearman ρ = 0.980–0.998). Bootstrap resampling further confirmed the stability of the framework (original Spearman ρ = 0.998, bootstrap bias = −0.0053, standard error = 0.0041, 95% percentile confidence interval = 0.983–0.999).

## 3. Discussion

Colorectal carcinoma arises from well-defined precancerous lesions known as conventional or serrated adenomas. Although both pathways—classical and serrated—leading to their formation are already well described, many unanswered questions remain regarding ITH in colorectal adenomas; for example, whether it varies in different parts of the colon or how it is related to specific histological types of adenomas.

Several interesting facts emerged from the general determination of mutation prevalence in our set of lesions. Consistent with the known key role of *BRAF*-activated mutation in the serrated pathway of colorectal carcinogenesis [10,19,52], our set of 43 serrated lesions (9 HPP and 34 TSA) contained *BRAF* mutation in the vast majority of cases (36/43, 84%). Furthermore, since it was present in only 7/141 (5%) of conventional adenomas, *BRAF* mutation represents a highly specific marker of serrated lesions, with a sensitivity of 84% and a specificity of 95%. *KRAS* mutation, which is also frequently associated with serrated lesions, was detected in 1/9 (11%) HPPs and 4/34 (12%) TSAs in our cohort. This frequency is lower than that reported in a study detecting *KRAS* mutations in 7/17 (41%) TSAs [53], but higher than in another study reporting the occurrence of KRAS mutations in 0/2 (0%) TSAs and 2/31 (6%) HPPs [54].

Our results also do not correspond to the previously predicted mutational exclusivity between *BRAF* and *KRAS* [10,55]. In our cohort, we recorded their simultaneous occurrence in 3 TSAs and 4 TVAs. This finding corresponds to previous observations, where the co-occurrence of both mutations within a single serrated lesion was also described [56]. Thanks to the comprehensive ITH analysis enabled by the newly introduced WTHT methodology, which allows detailed assessment of ITH throughout the entire adenoma, it was possible to localize both mutational clones. Their spatial distribution in all seven cases indicates the presence of *KRAS* and *BRAF* mutations in different cell clones (results are shown in Supplementary Table S4 and Supplementary Charts).

Another interesting finding concerns *TP53* mutations, which were detected in all histological types of precancerous colorectal lesions. Their frequency increased with the degree of dysplasia, and their highest incidence was observed in TVA. These findings may support a role of *TP53* mutations in adenoma progression and are consistent with the established role of *TP53* as a late event in colorectal carcinogenesis associated with increasing genomic instability and malignant potential [11,57]. Moreover, in our previous study, *TP53* mutations were associated with early recurrence of colorectal lesions [58], suggesting that their presence may also have prognostic significance. Taken together, these observations indicate that *TP53* mutations could represent a potential biomarker of increased biological aggressiveness and a predictor of higher risk of progression or recurrence of precancerous colorectal lesions.

The WTHT method is characterized in particular by systematic sampling of the complete tumor mass. For comparison with WTHT, the sampling methodologies previously used for ITH studies were approximated into three mathematical models, demonstrating that the method of tumor tissue sampling fundamentally influences the evaluation and interpretation of ITH. The approach based on homogenization of the entire tumor mass (1) provides an overview of the overall mutation spectrum, but loses information about the spatial localization and evolution of individual mutational clones, which corresponds with the general limits of bulk sequencing [59]. Another sampling strategy, based on a macrodissection of a limited number of larger sections (2), appears to be the least suitable for studying ITH, as it is strongly affected by tissue selection and may lead to an underestimation of the true level of heterogeneity, as previously described in connection with sampling bias in solid tumors [58]. In contrast, an approach based on sampling from multiple sites of the tumor combined with multiregional sequencing (3) already better reflects clonal diversity and enables the capture of tumor evolutionary dynamics [60,61]; however, it still does not provide a complete picture of ITH in the entire tumor.

The proposed WTHT method, based on the systematic division of the entire tumor mass into small-volume units followed by individual DNA analysis, enables accurate reconstruction of the spatial localization of mutational clones, their quantification in different tumor regions, and thus the determination of variance across the entire tumor. These findings are also reflected by the results of HG quantifying ITH (Table 1), where HG3 showed the closest results to HG4, while HG2 showed the greatest deviation from HG4. These results emphasize that for the accurate assessment of ITH, not only is the range of analyzed genetic markers crucial, but also, above all, the sampling strategy, which fundamentally affects the interpretation of ITH.

Although WTHT (HG4) provides the most comprehensive assessment of ITH, its implementation requires systematic processing of the entire lesion and analysis of a large number of samples, which may limit its applicability in routine practice. In contrast, the HG3 approach, based on random sampling of one region from each approximately 3 mm FFPE section, achieved the closest agreement with HG4 while requiring substantially fewer analyses. Therefore, HG3 could potentially be the preferred sampling strategy for routine molecular assessment of ITH in colorectal precancerous lesions.

At the same time, this study shows that variance—i.e., differences in the spatial distribution of individual mutational clones—is an important metric for assessing ITH. Its accurate determination requires sampling from as many regions of the tumor mass as possible, ideally by dividing the entire lesion volume into samples, as demonstrated in the WTHT method. Underestimation of ITH due to limited or locally selective sampling (e.g., from high-grade areas only) not only leads to distortion of its quantification but can, for example, mask the presence of minor mutational clones, which may represent a key source of tumor evolution, therapeutic resistance, and subsequent disease progression.

## 4. Materials and Methods

### 4.1. Patients and Samples

This study included 147 patients undergoing index colonoscopy at the Department of Gastrointestinal Endoscopy, Military University Hospital, Prague, Czech Republic, who met the two following entry criteria: 1. presence of at least one colorectal lesion larger than 1 cm (advanced lesion according to ESGE size criteria) at index colonoscopy, 2. removal of the lesion en bloc (in one whole piece) by endoscopic polypectomy, endoscopic mucosal resection, or endoscopic submucosal dissection. Written consent to participate in the study was obtained from all patients, which included a clause on the anonymization of samples and their use for scientific purposes only.

Thus, 184 index colorectal lesions were included in the study. Nineteen patients presented with multiple lesions, whose independence was verified by intra-patient pairwise similarity analysis using the Jaccard similarity index, viz. Supplementary Table S3. The lesions were vertically cut into 2–13 sections at the Department of Pathology, Military University Hospital, Prague, Czech Republic, fixed with formalin, and embedded in paraffin (FFPE). The histological type of the lesion and grade of dysplasia were determined, and finally the blocks, along with precise specifications of their spatial orientation in the adenoma, were delivered to the laboratories of Genomac Research Institute for mutation analysis.

### 4.2. Molecular Genetic Analysis

For best capture of ITH, FFPE blocks were further divided into smaller samples that were of equal size within each lesion and corresponded to approximately 10 mm^3^ of tissue, resulting in 3–58 samples in total from each lesion. DNA was isolated from each sample separately using the CatchGene™ FFPE Tissue DNA Kit (CatchGene, Taiwan), following the manufacturer’s instructions until tissue lysis. Due to the large number of samples, sample isolation was completed with a Zybio EXM3000 automated isolator (Zybio Inc., Chongqing, China) based on the principle of DNA capture on magnetic beads using the BIOBASE extraction kit (BIOBASE Group, Jinan, China) and the xTrovert™ FLEX-O™ robotic pipettor (Watrex Praha s.r.o., Prague, Czech Republic). DNA concentration was quantified using the QubitTM dsDNA BR Assay Kit (Thermo Fisher Scientific, Waltham, MA, USA), and DNA was finally diluted to a working concentration of 15 ng/µL.

DNA obtained from every sample was subjected to somatic mutation analysis of 11 target hotspot mutation regions characteristic of colorectal cancer. Specifically, it was the mutation cluster region in exon 15 of the *APC* gene (consisting of 4 PCR products *APC* I–IV), *TP53* exons 5–8 (*TP53*-5, *TP53*-6, *TP53*-7, *TP53*-8), *KRAS* exon 2, *PIK3CA* exon 9, and *BRAF* exon 15. The principle of the methodology consists in the amplification of target gene regions using PCR with heteroduplex formation and heteroduplex analysis at an optimal temperature using denaturing capillary electrophoresis on an ABI Prism 3730 XL instrument (Applied Biosystems, Waltham, MA, USA). All PCR reactions were performed in a 96-well plate under standardized laboratory protocol using previously optimized assay conditions for each analyzed amplicon. The sensitivity in detecting minor alleles ranges from 0.03% to 1% depending on the target region, and the specificity is 100% [62].

The electropherograms resulting from heteroduplex analysis were analyzed using GeneMarker v2.4.2 following automatic baseline correction. If any mutation was identified in any given sample of the lesion, peak heights were determined from the y-coordinate of each electrophoretic peak apex and used for PMF calculation according to Equation (1):

(1) $$PMF=\;\frac{A_{\mathrm{hom}MUT}+\;\frac{A_{het1}+A_{het2}}{2}}{A_{\mathrm{hom}MUT}+A_{\mathrm{hom}WT}+A_{het1}+A_{het2}}\cdot 100[\%],$$

where *A_hom MUT_* represents the peak height of the homoduplex of the mutant allele, *A_homWT_* the homoduplex of the wild type allele, and *A_het_*_1_ and *A_het_*_2_ the two heteroduplex peaks. Since both heteroduplexes contain one copy of the mutated allele and one copy of the wild type allele, each of them contributes one half to the mutant faction. Multiplying by 100 converts the ratio of the mutated fraction to the total number of alleles into a percentage. The workflow is shown in Figure 3.

### 4.3. Statistical Analysis

The presence of mutations in the *APC*, *KRAS*, *BRAF*, *PIK3CA,* and *TP53* genes was assessed depending on the anatomical localization (left colon, right colon, rectum) and histological type of the lesion (HPP, TSA, TA LGD, TA HGD, TA HGD ca, TVA LGD, TVA HGD, TVA HGD ca). A lesion was classified as mutated if a mutation in the respective gene (for *APC* and *TP53* in any analyzed region) was present in any of its samples. Mutation prevalence was expressed as the percentage of mutated lesions together with 95% Wilson confidence intervals. Differences in mutation prevalence between anatomical and histological groups were evaluated using Fisher’s exact test with Monte Carlo simulation (10,000 replicates). *p*-values were adjusted for multiple testing using the Benjamini–Hochberg false discovery rate procedure.

All data analysis and visualization were performed in R v4.5.1 (R Foundation for Statistical Computing, Vienna, Austria) using the tidyverse and ggplot2 packages. Heatmaps were generated using the Highcharts library v11 (Highsoft AS, Norway). Two- and three-dimensional spatial maps of *KRAS* and *BRAF* distribution were generated in R based on the original sampling scheme of each lesion, with PMF values displayed according to their spatial location within the lesion. Interactive three-dimensional visualizations were created using the Plotly v4,11,0 package.

Statistical analysis was also used to compare three modeled sampling approaches with our sampling approach; more specifically, to compare HG 1–3 values obtained using the respective modeled sampling approach with our HG4. For this purpose, bias, absolute and relative error, and distortion were calculated in R. Paired comparisons were performed using the Wilcoxon signed-rank test with Benjamini–Hochberg correction for multiple comparisons. The robustness of bias and error estimates was assessed by non-parametric bootstrap resampling (10,000 iterations), from which 95% confidence intervals were calculated. The calculation of HG is described below.

### 4.4. Differentiation Index and Heterogeneity Grade

For each mutation, the variance of PMF values across the lesion was calculated using sample variance. Then the distribution of variance values across the dataset was partitioned into five intervals of approximately equal frequency (quintile-based binning). The resulting intervals were defined as: 0–17.5, 17.5–37.8, 37.8–95.9, 95.9–200.1, and ≥200.1. Each interval was then assigned a Differentiation Index (DI) corresponding to increasing levels of intratumor PMF variance (0.6, 1.2, 1.8, 2.4, and 3.0, respectively). Quintile-based binning was chosen to ensure representation of the observed variance distribution while preserving the ordinal relationship between increasing variance and DI values.

Finally, the Heterogeneity Grade (HG) was calculated according to equation 2, combining the contribution of the spatial heterogeneity of individual mutations with the overall mutational complexity of the lesion. The mutational count term (2m) was designed so that the presence of an additional independent mutation contributes more strongly to *HG* than an increase in the variance category of a single mutation, based on the biological rationale that lesions harboring multiple independent clones are expected to represent greater intratumor complexity than lesions containing a single highly variable clone.

(2) $$HG=\sum_{j=1}^{m}{DI}_{j}+2m,$$

where *m* is the number of mutations and *DI_j_* is the Differentiation Index assigned to mutation *j* based on its variance interval. The robustness of the proposed *HG* framework was subsequently assessed by sensitivity analyses using alternative variance binning strategies (3, 4, 5, and 10 bins), different mutation-count weights (0, 1, 1.5, 2, and 3), and bootstrap validation.

### 4.5. Modelling of Sampling Strategies

HG calculations for the three other types of modeled sampling strategies listed in the Introduction (HG1–3) were performed:

HG1 was calculated for sampling strategy 1, which represented analysis of the entire lesion homogenized into a single sample. For each mutation found in a given lesion, the mean PMF value across all samples ($\bar{PMFg}$) was calculated as a quantitative indicator of the percentage of each mutated marker *g* within the entire lesion according to the following Equation (3):

(3) $$\bar{{PMF}_{g}}=\frac{1}{n_{g}}\sum_{i=1}^{n_{g}}{PMF}_{g,i}$$

As variance cannot be estimated from a single aggregated value, a constant differentiation index (*DI_j_* = 0.6) was assigned to all mutations.

The second modeled sampling strategy for calculating HG2 consisted of three sections, which in our dataset corresponded to tissue from approximately one FFPE section. Because each FFPE section in our dataset consisted of multiple smaller samples, PMF values were first aggregated within the FFPE section to obtain $\bar{PMFg}$ and all mutations were then assigned a constant DI of 0.6, as in the first sampling model.

HG3 calculation was used in the simulation of the third sampling strategy, the multiple sampling model, where one region was randomly selected from each FFPE section.

## 5. Conclusions

Our results demonstrate that the assessment of ITH is strongly influenced by the spatial sampling strategy used for molecular analysis. WTHT represents a shift from conventional approaches based on limited and often random tissue sampling toward systematic and spatially defined mapping of the entire tumor. By reducing the sampling bias associated with previous approaches, WTHT provides a new methodology for systematic spatial assessment of ITH, offering a potential tool for monitoring mutation development in precancerous lesions based on changes in key driver mutations.

## Figures and Tables

**Table 1:** Comparison of heterogeneity grades (HG) obtained using four sampling strategies. HG1 was calculated from a single representative tissue sample, HG2 from aggregated samples obtained from one representative FFPE section, HG3 from a multiple-sampling strategy using one randomly selected region from each FFPE section, and HG4 from complete WTHT sampling of the entire lesion, representing the reference estimate of ITH.

Lesion ID	Histology	HG_1_	HG_2_	HG_3_	HG_4_
**9**	HPP	2.6	2.6	3.8	3.2
		*BRAF*	*BRAF*	*BRAF* (var = 49)	*BRAF* (var = 22)
**13**	TVA LGD	7.8	2.6	9.6	12
		*KRAS* *TP53-5* *APC I*	*–* *–* *APC I*	*KRAS* (var = 4) *TP53-5* (var = 189) *APC I* (var = 10)	*KRAS* (var = 2) *TP53-5* (var = 134) *APC I* (var = 251)
**22**	TVA LGD	5.2	2.6	2.6	6.4
		*KRAS* *PIK3CA*	*KRAS* *–*	*KRAS* (var = 9) *–*	*KRAS* (var = 53) *PIK3CA* (var = 5)
**30**	TSA	13	10.4	17	19
		*KRAS* *BRAF* *PIK3CA* *TP53-6* *APC III*	*KRAS* *BRAF* *PIK3CA* *–* *APC III*	*KRAS* (var = 26) *BRAF* (var = 156) *PIK3CA* (var = 178) *–* *APC III* (var = 292)	*KRAS* (var = 25) *BRAF* (var = 79) *PIK3CA* (var = 122) *TP53-6* (var = 38) *APC III* (var = 160)
**36**	HPP	2.6	2.6	2.6	3.2
		*BRAF*	*BRAF*	*BRAF* (var = 12)	*BRAF* (var = 29)
**38**	TVA HGD	5.2	5.2	8.2	7
		*KRAS* *TP53-8*	*KRAS* *TP53-8*	*KRAS* (var = 51) *TP53-8* (var = 161)	*KRAS* (var = 32) *TP53-8* (var = 97)
**39**	TSA	13	13	17.2	17.2
		*BRAF* *TP53-5* *TP53-7* *TP53-8* *APC III*	*BRAF* *TP53-5* *TP53-7* *TP53-8* *APC III*	*BRAF* (var = 169) *TP53-5* (var = 36) *TP53-7* (var = 56) *TP53-8* (var = 17) *APC III* (var = 22)	*BRAF* (var = 194) *TP53-5* (var = 40) *TP53-7* (var = 26) *TP53-8* (var = 15) *APC III* (var = 21)
**43**	TVA HGD (ca)	7.8	7.8	8.2	12.6
		*KRAS* *BRAF* *APC III*	*KRAS* *BRAF* *APC III*	*KRAS* (var = 165) *–* *APC III* (var = 86)	*KRAS* (var = 275) *BRAF* (var = 7) *APC III* (var = 247)
**48**	TVA LGD	5.2	2.6	7.6	7
		*KRAS* *TP53-5*	*–* *TP53-5*	*KRAS* (var = 56) *TP53-5* (var = 64)	*KRAS* (var = 27) *TP53-5* (var = 53)
**74**	TSA	10.4	7.8	14.6	13.4
		*BRAF* *TP53-5* *TP53-7* *TP53-8*	*BRAF* *TP53-5* *–* *TP53-8*	*BRAF* (var = 321) *TP53-5* (var = 135) *TP53-7* (var = 16) *TP53-8* (var = 2)	*BRAF* (var = 143) *TP53-5* (var = 86) *TP53-7* (var = 5) *TP53-8* (var = 3)
**80**	TVA HGD (ca)	2.6	2.6	4.4	5
		*KRAS*	*KRAS*	*KRAS* (var = 96)	*KRAS* (var = 308)
**165**	TA LGD	5.2	2.6	4.4	9.4
		*KRAS* *APC IV*	*KRAS* *–*	*KRAS* (var = 114) *–*	*KRAS* (var = 121) *APC IV* (var = 271)
**167**	TVA HGD	2.6	–	4.4	5
		*KRAS*	*–*	*KRAS* (*var* = 146)	*KRAS* (*var* = 220)
**174**	TVA LGD	5.2	2.6	5	7.6
		*KRAS* *TP53-8*	*KRAS* *–*	*KRAS* (*var* = 456) *–*	*KRAS* (*var* = 115) *TP53-8* (*var* = 36.27)
**180**	TVA HGD	13	7.8	15.2	18.4
		*KRAS* *BRAF* *APC III* *APC IV* *APC VII*	*KRAS* *–* *–* *APC IV* *APC VII*	*KRAS* (var = 44) *–* *APC III* (var = 5) *APC IV* (var = 531) *APC VII* (var = 46)	*KRAS* (var = 145) *BRAF* (var = 5) *APC III* (var = 5) *APC IV* (var = 381) *APC VII* (var = 47)
**197**	TA HGD (ca)	2.6	2.6	4.4	3.8
		*TP53-8*	*TP53-8*	*TP53-8* (var = 181)	*TP53-8* (var = 83)
**206**	TA LGD	5.2	2.6	4.4	8.8
		*KRAS* *APC III*	*–* *APC III*	*–* *APC III* (var = 114)	*KRAS* (var = 179) *APC III* (var = 135)
**236**	TVA LGD	10.4	7.8	16.4	15.8
		*KRAS* *TP53-8* *APC III* *APC VII*	*–* *TP53-8* *APC III* *APC VII*	*KRAS* (var = 15) *TP53-8* (var = 344) *APC III* (var = 54) *APC VII* (var = 298)	*KRAS* (var = 15) *TP53-8* (var = 344) *APC III* (var = 54) *APC VII* (var = 240)
**237**	TVA LGD	7.8	7.8	14.4	12.6
		*KRAS* *TP53-5* *APC IV*	*KRAS* *TP53-5* *APC IV*	*KRAS* (var = 220) *TP53-5* (var = 103) *APC IV* (var = 320)	*KRAS* (var = 139) *TP53-5* (var = 64) *APC IV* (var = 183)
**246**	TA HGD (ca)	5.2	5.2	8.8	10
		*KRAS* *TP53-8*	*KRAS* *TP53-8*	*KRAS* (var = 108) *TP53-8* (var = 175)	*KRAS* (var = 206) *TP53-8* (var = 450)

var = variance.

## Data Availability

The original contributions presented in this study are included in the article/Supplementary Material. Further inquiries can be directed to the corresponding author.

## Supplementary Materials

The following supporting information can be downloaded at: https://www.mdpi.com/article/10.3390/ijms27156547/s1.

## Abbreviations

The following abbreviations are used in this manuscript:

WTHT	Whole Tumor Heterogeneity Topography
ITH	Intratumor Heterogeneity
HG	Heterogeneity Grade
PMF	Proportion of Mutant Fraction
