# Supplementary material for: Whole Tumor Heterogeneity Topography (WTHT)—A New Approach for Assessment of Intratumor Mutational Heterogeneity in Colorectal Adenomas
Source: Int J Mol Sci. 2026 Jul 23;27(15):6547. doi: 10.3390/ijms27156547 (PMC13465348; doi:10.3390/ijms27156547)
Supplement: Supplementary file 1 [file ijms-27-06547-s001.zip › ijms-4379229-supplementary Figure S1.pdf]

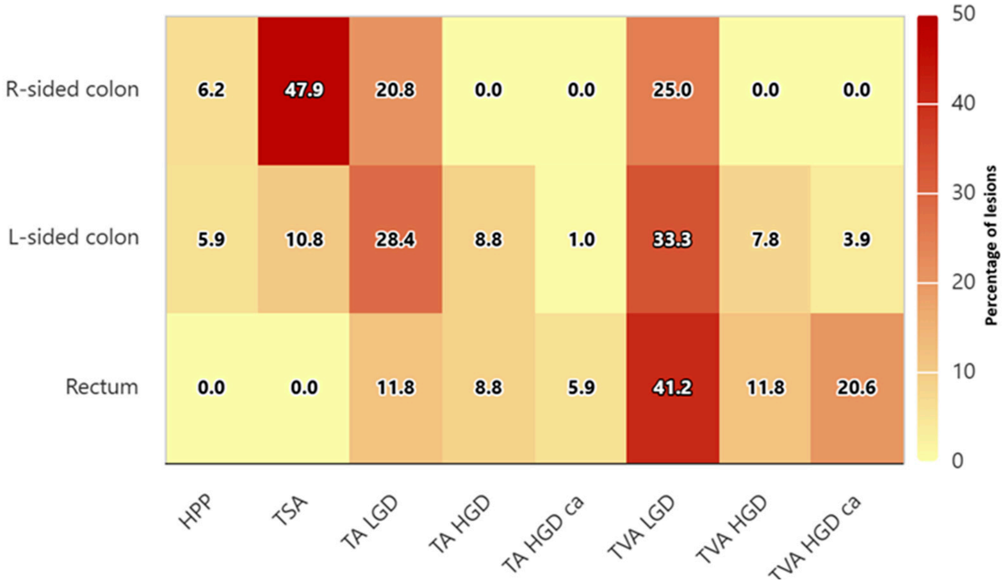

Supplementary Figure S1. The incidence of histological types of colorectal lesions depending on their localizations. Heatmap shows the relative incidence (%) of individual histological types of colorectal lesions in different parts of the colon (right-sided colon, left-sided colon, and rectum).
