# Supplementary material for: Whole Tumor Heterogeneity Topography (WTHT)—A New Approach for Assessment of Intratumor Mutational Heterogeneity in Colorectal Adenomas
Source: Int J Mol Sci. 2026 Jul 23;27(15):6547. doi: 10.3390/ijms27156547 (PMC13465348; doi:10.3390/ijms27156547)
Supplement: Supplementary file 1 [file ijms-27-06547-s001.zip › Supplementary_table_S2.pdf]

Prevalence of mutations according to anatomical localisation with 95% Wilson confidence intervals

Gene	Localisation	Positive/total	Prevalence [%]	95% CI
KRAS	Left-sided colon	30/102	29.4	21.4–38.9
	Right-sided colon	11/48	22.9	13.3–36.5
	Rectum	21/34	61.8	45.0–76.1
BRAF	Left-sided colon	14/102	13.7	8.4–21.7
	Right-sided colon	25/48	52.1	38.3–65.5
	Rectum	4/34	11.8	4.7–26.6
TP53	Left-sided colon	21/102	20.6	13.9–29.4
	Right-sided colon	5/48	10.4	4.5–22.2
	Rectum	12/34	35.3	21.5–52.1
APC	Left-sided colon	26/102	25.5	18.0–34.7
	Right-sided colon	6/48	12.5	5.9–24.7
	Rectum	6/34	17.6	8.3–33.5
PIK3CA	Left-sided colon	3/102	2.9	1.0–8.3
	Right-sided colon	1/48	2.1	0.4–10.9
	Rectum	4/34	11.8	4.7–26.6

Prevalence of mutations according to histological group with 95% Wilson confidence intervals

Gene	Histological group	Positive/total	Prevalence [%]	95% CI
KRAS	HPP	1/9	11.1	2.0–43.5
	TSA	4/34	11.8	4.7–26.6
	TA LGD	7/43	16.3	8.1–30.0
	TA HGD	5/12	41.7	19.3–68.0
	TA HGD (ca)	2/3	66.7	20.8–93.9
	TVA LGD	30/60	50.0	37.7–62.3
	TVA HGD	8/12	66.7	39.1–86.2
	TVA HGD (ca)	5/11	45.5	21.3–72.0
BRAF	HPP	7/9	77.8	45.3–93.7
	TSA	24/34	70.6	53.8–83.2
	TA LGD	0/43	0.0	0.0–8.2
	TA HGD	0/12	0.0	0.0–24.2
	TA HGD (ca)	0/3	0.0	0.0–56.1
	TVA LGD	4/60	6.7	2.6–15.9
	TVA HGD	1/12	8.3	1.5–35.4
	TVA HGD (ca)	2/11	18.2	5.1–47.7
PIK3CA	HPP	0/9	0.0	0.0–29.9
	TSA	1/34	2.9	0.5–14.9

	TA LGD	2/43	4.7	1.3–15.5
	TA HGD	0/12	0.0	0.0–24.2
	TA HGD (ca)	0/3	0.0	0.0–56.1
	TVA LGD	4/60	6.7	2.6–15.9
	TVA HGD	1/12	8.3	1.5–35.4
	TVA HGD (ca)	0/11	0.0	0.0–25.9
TP53	HPP	1/9	11.1	2.0–43.5
	TSA	6/34	17.6	8.4–33.8
	TA LGD	5/43	11.6	5.1–24.2
	TA HGD	2/12	16.7	4.7–44.8
	TA HGD (ca)	2/3	66.7	20.8–93.9
	TVA LGD	23/60	38.3	27.1–50.9
	TVA HGD	6/12	50.0	25.4–74.6
	TVA HGD (ca)	8/11	72.7	43.4–90.3
APC	HPP	0/9	0.0	0.0–29.9
	TSA	4/34	11.8	4.7–26.6
	TA LGD	9/43	20.9	11.3–35.4
	TA HGD	1/12	8.3	1.5–35.6
	TA HGD (ca)	1/3	33.3	6.1–79.2
	TVA LGD	17/60	28.3	18.5–40.9
	TVA HGD	2/12	16.7	4.7–44.8
	TVA HGD (ca)	2/11	18.2	5.1–47.7

Statistical comparison of mutation prevalence between histological groups

Gene	Fisher’s exact test P	Benjamini–Hochberg adjusted P
KRAS	0.00010	0.00025
BRAF	0.00010	0.00025
PIK3CA	0.58774	0.58774
TP53	0.18178	0.22723
APC	0.13779	0.22723

Statistical comparison of mutation prevalence between anatomical locations

Gene	Fisher’s exact test P	Benjamini–Hochberg adjusted P
KRAS	0.00050	0.00125
BRAF	0.00010	0.00050
PIK3CA	0.08289	0.10361
TP53	0.02160	0.03600
APC	0.18218	0.18218
