# Supplementary material for: Whole Tumor Heterogeneity Topography (WTHT)—A New Approach for Assessment of Intratumor Mutational Heterogeneity in Colorectal Adenomas
Source: Int J Mol Sci. 2026 Jul 23;27(15):6547. doi: 10.3390/ijms27156547 (PMC13465348; doi:10.3390/ijms27156547)
Supplement: Supplementary file 1 [file ijms-27-06547-s001.zip › Supplementary_table_S3.pdf]

Characteristics of patients with multiple colorectal lesions

| Patient | Lesion | localisation | histology | mutations |
|---|---|---|---|---|
| 1 | I | left-sided colon | TVA LGD | |
| | II | left-sided colon | TVA HGD | *KRAS* |
| 2 | I | left-sided colon | HPP | *BRAF* |
| | II | left-sided colon | HPP | *BRAF* |
| | III | left-sided colon | TVA HGD | *TP53-7* |
| | IV | left-sided colon | TVA LGD | *APC III* |
| 3 | I | right-sided colon | TSA | *BRAF* |
| | II | right-sided colon | TSA | *BRAF* |
| | III | right-sided colon | TSA | *BRAF* |
| | IV | right-sided colon | TSA | *KRAS* |
| 4 | I | left-sided colon | TA LGD | |
| | II | left-sided colon | TA LGD | |
| | III | left-sided colon | TSA | *KRAS; BRAF; PIK3CA; TP53-6; APC III* |
| 5 | I | right-sided colon | TA LGD | |
| | II | left-sided colon | TA LGD | *TP53-7* |
| 6 | I | left-sided colon | TVA LGD | |
| | II | left-sided colon | HPP | *BRAF* |
| | III | left-sided colon | TVA LGD | |
| | IV | rectum | TA LGD | |
| | V | rectum | TVA LGD | *TP53-6* |
| 7 | I | left-sided colon | TVA LGD | *KRAS* |
| | II | left-sided colon | TVA HGD | |
| 8 | I | right-sided colon | TSA | *KRAS; BRAF; TP53-5; TP53-8* |
| | II | right-sided colon | TSA | *BRAF* |
| | III | left-sided colon | TVA HGD (ca) | *KRAS* |
| 9 | I | left-sided colon | TVA HGD | *KRAS; BRAF; APC III* |
| | II | left-sided colon | TVA LGD | *KRAS* |
| 10 | I | right-sided colon | TA LGD | *KRAS; APC IV* |
| | II | left-sided colon | TVA LGD | |
| | III | left-sided colon | TVA HGD | *KRAS* |
| 11 | I | left-sided colon | TVA LGD | *PIK3CA* |
| | II | left-sided colon | TVA LGD | *KRAS; TP53-8* |
| 12 | I | right-sided colon | TVA LGD | |
| | II | rectum | TVA HGD | *KRAS; BRAF; APC III; APC IV; APC VII* |
| 13 | I | right-sided colon | HPP | |
| | II | rectum | TA LGD | |
| 14 | I | left-sided colon | TSA | *BRAF* |
| | II | left-sided colon | TA LGD | |
| 15 | I | left-sided colon | TA HGD | *KRAS* |
| | II | left-sided colon | TA LGD | *APC III* |
| | III | left-sided colon | TA LGD | *KRAS; APC III* |
| | IV | left-sided colon | TA HGD | *KRAS* |
| 16 | I | left-sided colon | TA LGD | |
| | II | left-sided colon | TA LGD | *APC III* |
| 17 | I | left-sided colon | TA LGD | *TP53-5* |
| | II | left-sided colon | TA LGD | |
| | III | left-sided colon | TA LGD | *TP53-5* |
| | IV | right-sided colon | TSA | *BRAF* |
| | V | left-sided colon | TA LGD | |
| 18 | I | left-sided colon | TSA | *BRAF* |
| | II | left-sided colon | TSA | *BRAF* |
| 19 | I | right-sided colon | HPP | *BRAF* |
| | II | right-sided colon | TSA | *BRAF; APC I* |

Summary of intra-patient pairwise similarity analysis.

| Parameter | Result |
|---|---|
| Mean Jaccard similarity | 0.20 |
| Median Jaccard similarity | 0.00 |
| Lesion pairs sharing no mutation | 44/59 (74.6%; 95% CI 62.2–83.9%) |
| Lesion pairs sharing no non-contextual mutation* | 51/59 (86.4%; 95% CI 75.5–93.0%) |
| Different histology | 35/59 (59.3%; 95% CI 46.6–70.9%) |
| Different histological group | 24/59 (40.7%; 95% CI 29.1–53.4%) |
| Different dysplasia grade | 29/59 (49.2%; 95% CI 36.8–61.6%) |
| Different anatomical localisation | 17/59 (28.8%; 95% CI 18.8–41.4%) |

Concordant BRAF mutations in serrated lesions (HPP/TSA) were regarded as recurrent lineage-associated events rather than evidence of clonal relatedness and were therefore excluded from the corrected similarity analysis.
