# Supplementary material for: Whole Tumor Heterogeneity Topography (WTHT)—A New Approach for Assessment of Intratumor Mutational Heterogeneity in Colorectal Adenomas
Source: Int J Mol Sci. 2026 Jul 23;27(15):6547. doi: 10.3390/ijms27156547 (PMC13465348; doi:10.3390/ijms27156547)
Supplement: Supplementary file 1 [file ijms-27-06547-s001.zip › Supplementary_table_S4.pdf]

Lesion ID	Lesion sample	*KRAS*	*BRAF*
30<br>TSA	1a		28.45%
	1b		5.95%
	1c		
	2a	4.94%	2.46%
	2b		18.30%
	2c		
	3a		6.81%
	3b		3.75%
	3c	10.26%	
	4a		
	4b	14.02%	
35<br>TSA	1a		26.54%
	1b		19.26%
	1c		18.98%
	2a		26.95%
	2b		20.51%
	3a		
	3b		16.82%
	4a	2.53%	
	4b		
43<br>TVA HGD (ca)	1a		
	1b		
	1c		
	1d		
	1e	21.69%	
	1f	34.20%	
	1g		
	2a		
	2b	9.39%	
	2c	14.74%	
	2d		
	2e		
	2f	9.60%	
	2g		
	2h	15.81%	
	2i		
	3a		
	3b		
	3c	28.16%	
	3d		
	3e		
	3f		
	3g	15.07%	
	3h		
	3i		
	4a	47.83%	
	4b	34.28%	
	4c		12.54%

	4d		8.87%
	4e	49.60%	
	4f		
	4g	15.32%	
	4h		
	5a		
	5b		
	5c	38.24%	
	5d	44.50%	
	5e	24.02%	
	5f		
	5g	23.27%	
	6a		
	6b		
	6c		
	6d	41.17%	
	6e		
	6f	12.57%	
	6g	26.18%	
78<br>TSA	1a		13.70%
	1b	42.13%	6.70%
	2a		11.97%
	2b		19.06%
	3a		7.25%
	3b		14.67%
	3c		8.30%
	4a		
	4b		
	4c		7.98%
	5a		
	5b		
88<br>TVA LGD	1a		4.84%
	1b		
	1c		
	1d		
	2a		
	2b		8.75%
	2c	5.53%	
	2d	16.15%	
	3a	16.68%	
	3b		
	3c	10.79%	
	3d	13.89%	
	4a	12.33%	
	4b		
	4c	17.91%	
	4d		
180<br>TVA HGD	1a	44.62%	
	1b	38.57%	

	1c	27.77%	11.35%
	1d	33.25%	6.37%
	1e	41.76%	
	2a		
	2b		
	2c	44.14%	
	2d	41.91%	
	2e	42.59%	
	3a	40.13%	
	3b	33.87%	
	3c	44.41%	
	3d	46.34%	
	4a	48.40%	
	4b	40.67%	
	4c	44.31%	
	4d	42.80%	
	4e	42.20%	
	4f	44.76%	
	5a	40.12%	
	5b	48.83%	
	5c		
	5d	46.02%	
	5e	42.31%	
	6a	45.83%	
	6b	44.06%	
	6c	47.07%	
	6d	45.79%	
	7a	38.07%	
	7b	44.21%	
	7c	40.79%	
	7d	43.12%	
	8a	50.21%	
	8b	42.62%	
	8c	42.81%	
	8d	50.81%	
	9a	40.94%	
	9b		
	9c	46.75%	
	9d	46.19%	
	10a	44.87%	
	10b	43.84%	
	10c	48.81%	
	10d	46.45%	
	11a	20.23%	
	11b		
	11c	37.85%	
	11d	31.63%	
250<br>TVA LGD	1a		
	1b		

	1c	13.17%	
	1d	15.44%	
	1e	11.00%	
	1f		
	2a		
	2b		
	2c	14.12%	
	2d	29.42%	
	2e	16.13%	
	2f		
	3a		
	3b		
	3c		
	3d		
	3e	9.69%	
	3f	10.17%	
	4a	13.69%	
	4b		
	4c		6.62%
	4d	11.40%	22.25%
	4e		
	4f		
	5a		
	5b		
